# Supplementary material for: Non-Pharmacological Preventive Measures Had an Impact on COVID-19 in Healthcare Workers before the Vaccination Effect: A Cohort Study
Source: Int J Environ Res Public Health. 2022 Mar 18;19(6):3628. doi: 10.3390/ijerph19063628 (PMC8955756; doi:10.3390/ijerph19063628)
Supplement: Supplementary file 1 [file ijerph-19-03628-s001.zip › ijerph-1641980-supplementary.pdf]

Supplementary Table

Table S1. Main sociodemographic and occupational characteristics. PSMar healthcare workers, 2020 (*n* = 5543).

		n	%
Sex	Women	4,066	73.4
	Men	1,477	26.6
Age	18-29 years	1,701	30.7
	30-49 years	2,401	43.3
	50-70 years	1,439	26.0
Health building centres	Hospital Mar	3,641	65.7
	Hospital Esperança	541	9.8
	Fòrum centre	450	8.1
	Dr. Emili Mira Centre	586	10.6
	Others	321	5.8
Contract arrangement	Permanent	3,358	61.7
	Temporary	1,106	20.0
	Replacement	1,017	18.3
Occupational category	Physicians	838	15.1
	Nurses and aides	2,832	51.1
	Other healthcare workers	1,005	18.1
	Administration and management staff	868	15.7
